# Drug–drug interaction and initial dosage optimization of aripiprazole in patients with schizophrenia based on population pharmacokinetics

**DOI:** 10.3389/fpsyt.2024.1377268

**Published:** 2024-06-18

**Authors:** Cun Zhang, Lei Jiang, Ke Hu, Yi-Jia Zhang, Jing Han, Jin Chen, Bulubu, Boling Dong, Hao-Zhe Shi, Su-Mei He, Ting-Ting Yu, Xiao Chen, Dong-Dong Wang

**Affiliations:** ^1^ Department of Pharmacy, Xuzhou Oriental Hospital Affiliated to Xuzhou Medical University, Xuzhou, Jiangsu, China; ^2^ Jiangsu Key Laboratory of New Drug Research and Clinical Pharmacy and School of Pharmacy, Xuzhou Medical University, Xuzhou, Jiangsu, China; ^3^ Department of Pharmacy, Taixing People’s Hospital, Taixing, Jiangsu, China; ^4^ National Demonstration Center for Experimental Basic Medical Science Education, Xuzhou Medical University, Xuzhou, Jiangsu, China; ^5^ Department of Pharmacy, Suzhou Hospital, Affiliated Hospital of Medical School, Nanjing University, Suzhou, Jiangsu, China; ^6^ School of Nursing, Xuzhou Medical University, Xuzhou, Jiangsu, China

**Keywords:** drug-drug interaction, initial dosage optimization, aripiprazole, patients with schizophrenia, population pharmacokinetics

## Abstract

**Background:**

The present study aimed to investigate the drug–drug interaction and initial dosage optimization of aripiprazole in patients with schizophrenia based on population pharmacokinetics.

**Research design and methods:**

A total of 119 patients with schizophrenia treated with aripiprazole were included to build an aripiprazole population pharmacokinetic model using nonlinear mixed effects.

**Results:**

The weight and concomitant medication of fluoxetine influenced aripiprazole clearance. Under the same weight, the aripiprazole clearance rates were 0.714:1 in patients with or without fluoxetine, respectively. In addition, without fluoxetine, for the once-daily aripiprazole regimen, dosages of 0.3 and 0.2 mg kg^−1^ day^−1^ were recommended for patients with schizophrenia weighing 40–95 and 95–120 kg, respectively, while for the twice-daily aripiprazole regimen, 0.3 mg kg^−1^ day^−1^ was recommended for those weighing 40–120 kg. With fluoxetine, for the once-daily aripiprazole regimen, a dosage of 0.2 mg kg^−1^ day^−1^ was recommended for patients with schizophrenia weighing 40–120 kg, while for the twice-daily aripiprazole regimen, 0.3 and 0.2 mg kg^−1^ day^−1^ were recommended for those weighing 40–60 and 60–120 kg, respectively.

**Conclusion:**

This is the first investigation of the effects of fluoxetine on aripiprazole via drug–drug interaction. The optimal aripiprazole initial dosage is recommended in patients with schizophrenia.

## Highlights

- Weight and concomitant medication of fluoxetine influenced aripiprazole clearance. Under the same weight, the aripiprazole clearance rates were 0.714:1 in patients with or without fluoxetine, respectively.
- Without fluoxetine, for the once-daily aripiprazole regimen, dosages of 0.3 and 0.2 mg kg^−1^ day^−1^ were recommended for patients with schizophrenia weighing 40–95 and 95–120 kg, respectively, while for the twice-daily aripiprazole regimen, 0.3 mg kg^−1^ day^−1^ was recommended for those weighing 40–120 kg.
- With fluoxetine, for the once-daily aripiprazole regimen, a dosage of 0.2 mg kg^−1^ day^−1^ was recommended for patients with schizophrenia weighing 40–120 kg, while for the twice-daily aripiprazole regimen, 0.3 and 0.2 mg kg^−1^ day^−1^ were recommended for those weighing 40–60 and 60–120 kg, respectively.
- This is the first study to investigate the effects of fluoxetine on aripiprazole via drug–drug interaction. The optimal aripiprazole initial dosage is recommended in patients with schizophrenia based on population pharmacokinetics. The aripiprazole dosage might need to be adjusted in patients with schizophrenia with concomitant medication of fluoxetine.

## 1 Introduction

Schizophrenia is a mental disorder characterized by hallucination and delusion, and its pathogenesis includes genetic and environmental factors. The prevalence of schizophrenia is approximately 0.3% in the world population (1, 2). Patients with schizophrenia are also accompanied with deficits in working memory, verbal fluency, and processing speed, among others (3). In addition, schizophrenia has serious impacts not only on patients but also on the society, e.g., increasing the social burden, triggering severe functional impairments, increasing the likelihood of disability, decreasing the quality of life, and reducing the life span of the affected patients (4). In clinical practice, the therapeutic goals for schizophrenia are to relieve symptoms, to prevent relapses, and to restore the functional capacities of schizophrenia patients (2, 5, 6). Of these, drug treatment is crucial (2).

Aripiprazole is a second-generation antipsychotic with low incidences of side effects and metabolic adverse events (7, 8); however, due to the characteristics of its pharmaceutical action, some researchers have considered aripiprazole a third-generation antipsychotic (2). Aripiprazole is a partial agonist with high affinity for the dopamine receptors R-D_2_ and R-D_3_ and the serotonin 1A receptor (R-5-HT_1A_), combined with an antagonistic activity on R-5-HT_2A_ (9). In addition, aripiprazole also has moderate affinity for R-5-HT_7_ (10) and R-5-HT_2C_, playing the role of a partial agonist (2).

Due to its lower incidence of side effects and its extensive pharmacological characteristics, aripiprazole is frequently chosen as the first-line therapeutic regimen for the first episode of psychosis, and it has exhibited acceptable efficacy (11, 12). Furthermore, schizophrenia treatment should be individualized based on the characteristics of each patient; however, the narrow therapeutic window and the considerable inter- and intra-individual pharmacokinetic variabilities (13) make formulating an aripiprazole initial dosage regimen for patients with schizophrenia difficult. At the same time, schizophrenia is often accompanied by the use of multiple drugs, and drug–drug interaction could also significantly affect the pharmacokinetic process of aripiprazole and its dosage optimization. Therefore, the present study aimed to investigate the drug–drug interaction and initial dosage optimization of aripiprazole in patients with schizophrenia based on population pharmacokinetics.

## 2 Methods

### 2.1 Data collection

Patients with schizophrenia treated with aripiprazole between July 2020 and June 2022 from the Xuzhou Oriental Hospital Affiliated to Xuzhou Medical University were included in the study and analyzed retrospectively. The aripiprazole concentrations were based on the Therapeutic Drug Monitoring (TDM) database. Related medical information was extracted from medical logs, including the physiological and biochemical indices (i.e., gender, age, weight, aripiprazole dosage form, albumin, globulin, alanine transaminase, aspartate transaminase, creatinine, urea, total protein, total cholesterol, triglyceride, direct bilirubin, total bilirubin, hematocrit, hemoglobin, mean corpuscular hemoglobin, and mean corpuscular hemoglobin concentration) and concomitant drugs. The research was approved by the Research Ethics Committee of the Xuzhou Oriental Hospital Affiliated to Xuzhou Medical University (no. 20230606005).

### 2.2 Modeling

The Nonlinear Mixed Effects Modeling (NONMEM, edition 7; ICON Development Solutions, Ellicott City, MD, USA) software with a first-order conditional estimation method with interaction (FOCE-I method) was used to build the aripiprazole population pharmacokinetic model, which included the pharmacokinetic parameters apparent oral clearance (CL/F), volume of distribution (*V*/F), and the absorption rate constant (*K*_a_, fixed at 1.06/h) (14).

Equation 1 describes the inter-individual variabilities.


(1) $$J_{i}=TV\left(J\right)\times exp\;\left(\eta_{i}\right)$$

where *J*_i_ is the individual parameter value; TV(*J*) is the typical individual parameter value; and *η_i_* is the random term with zero mean and variance omega^2 (*ω* ^2^).

Equation 2 describes the random residual variabilities.


(2) $$Q_{i}=P_{i}+P_{i}*\varepsilon_{1}+\varepsilon_{2}$$

where *Q*_i_ is the observed concentration and *P*_i_ is the individual predicted concentration. *ε*_1_ and *ε*_2_ are random terms with zero mean and variance sigma^2 (*σ* ^2^).

Equation 3 describes the association of the pharmacokinetic parameters with weight.


(3) $$W_{i}=W_{std}\times {\left(X_{i}/X_{std}\right)}^{R}$$

where *W_i_* is the *i*th individual parameter; *X_i_* is the *i*th individual weight; *X*_std_ is the standard weight of 70 kg; and *W*_std_ is a typical individual parameter whose weight is *X*_std_. *R* is the allometric coefficient: 0.75 for CL/F and 1 for *V*/F (15).

Equations 4, 5 describe the pharmacokinetic parameters between the continuous covariates and the categorical covariates, respectively.


(4) $$Y_{i}=TV\left(Y\right)\times {\left(Cov_{i}/Cov_{m}\right)}^{\theta }$$

(5) $$Y_{i}=TV\left(Y\right)\times \left(1+\theta \times Cov_{i}\right)$$

where *Y*_i_ is the individual parameter value; TV(*Y*) is the typical individual parameter value; *θ* is the parameter to be estimated; Cov*_i_* is the covariate of the *i*th individual; and Cov_m_ is the population median for the covariate.

The covariate model was established in a stepwise manner. Changes in the objective function value (OFV) were calculated as the covariate inclusion criteria, which included the processes of forward inclusion and backward elimination. A decrease of OFV >6.63 (*p* < 0.01) was defined as the forward inclusion, while an increase of OFV >10.8 (*p* < 0.001) was defined as the backward elimination.

### 2.3 Model evaluation

The final model was evaluated using observations vs. population predictions, the absolute value of the weighted residuals of individuals (│iWRES│) vs. individual predictions, observations vs. individual predictions, weighted residuals vs. time, density vs. weighted residuals, the quantiles of weighted residuals vs. the quantiles of normal, visual predictive check (VPC) of the model, and individual plots. In addition, the medians and the 2.5th–97.5th percentile ranges of the results from bootstraps (*n* = 1,000) were used for comparison with the final model parameters.

### 2.4 Simulation

The aripiprazole initial dosage optimization was executed using Monte Carlo simulation. Based on the report of Hart et al., the treatment window of aripiprazole for schizophrenia was 120–270 ng/ml (16). In the present study, it was found that the weight and the concomitant medication of fluoxetine influenced aripiprazole clearance. Thus, according to whether fluoxetine was used in combination, and a once-daily or a twice-daily aripiprazole regimen, the present study simulated four different scenarios, with each scenario including 1,000 virtual patients with schizophrenia, and eight dosages (i.e., 0.1, 0.2, 0.3, 0.4, 0.5, 0.6, 0.7, and 0.8 mg kg^−1^ day^−1^) for five weight groups (i.e., 40, 60, 80, 100, and 120 kg). The twice-daily aripiprazole regimen was split evenly into two dosages a day. The probability to achieve the target concentration window was selected as the evaluation criterion. The specific calculation process comprised in the number of patients reaching the treatment window compared with the simulated 1,000 virtual patients.

## 3 Results

### 3.1 Patient information

A total of 119 patients with schizophrenia were included for analysis, including 57 men and 62 women aged from 19.00 to 69.38 years. The demographic data of the patients and the drug combinations are shown in **Tables 1**, **2**.

**Table 1:** Demographic data of patients (*n* = 119).

Characteristic	Mean ± SD	Median (range)
Gender (men/women)	57/62	–
Age (years)	44.29 ± 13.03	46.84 (19.00–69.38)
Weight (kg)	66.77 ± 11.68	67.00 (41.00–115.00)
Aripiprazole dosage form (tablet/orally disintegrating tablet/solution)	95/35/1	12 patients used two dosage forms
Albumin (g/L)	41.96 ± 2.83	41.75 (33.70–50.40)
Globulin (g/L)	26.97 ± 3.73	26.60 (17.10–39.70)
Alanine transaminase (IU/L)	26.16 ± 19.36	19.00 (7.00–141.00)
Aspartate transaminase (IU/L)	22.71 ± 10.79	20.00 (10.00–96.00)
Creatinine (μmol/L)	64.41 ± 14.15	62.00 (4.03–112.00)
Urea (mmol/L)	5.74 ± 21.65	4.02 (1.27–324.00)
Total protein (g/L)	68.98 ± 4.57	68.90 (55.70–81.50)
Total cholesterol (mmol/L)	4.23 ± 0.88	4.16 (0.92–7.16)
Triglyceride (mmol/L)	1.60 ± 0.98	1.28 (0.56–5.90)
Direct bilirubin (μmol/L)	3.79 ± 1.79	3.30 (1.30–12.90)
Total bilirubin (μmol/L)	10.58 ± 4.89	9.50 (3.50–34.10)
Hematocrit (%)	39.50 ± 4.83	39.05 (29.90–53.10)
Hemoglobin (g/L)	130.28 ± 27.68	127.00 (93.00–459.00)
Mean corpuscular hemoglobin (pg)	30.29 ± 1.86	30.55 (21.70–35.30)
Mean corpuscular hemoglobin concentration (g/L)	326.20 ± 9.56	326.00 (296.00–356.00)

**Table 2:** Drug combinations.

Drug	Category	*N*	Drug	Category	*N*
Acarbose capsule	0	108	Fluvoxamine maleate tablet	0	116
	1	11		1	3
Alprazolam tablet	0	98	Levothyroxine sodium tablet	0	112
	1	21		1	7
Amisulpride tablet	0	115	Lorazepam tablet	0	103
	1	4		1	16
Aspirin enteric-coated tablet	0	114	Metformin hydrochloride tablet	0	104
	1	5		1	15
Atorvastatin calcium tablet	0	112	Metoprolol tartrate tablet	0	113
	1	7		1	6
Benzhexol hydrochloride tablet	0	82	Nifedipine sustained-release tablet	0	115
	1	37		1	4
Buspirone hydrochloride tablet	0	113	Olanzapine tablet	0	96
	1	6		1	23
Chlorpromazine hydrochloride tablet	0	118	Paliperidone extended-release tablet	0	115
	1	1		1	4
Clonazepam tablet	0	113	Paroxetine hydrochloride tablet	0	117
	1	6		1	2
Clozapine dispersible tablet	0	110	Propranolol hydrochloride tablet	0	96
	1	9		1	23
Clozapine tablet	0	77	Quetiapine fumarate tablet	0	114
	1	42		1	5
Compound captopril tablet	0	118	Risperidone tablet	0	105
	1	1		1	14
Docusate sodium tablet	0	112	Sertraline hydrochloride tablet	0	116
	1	7		1	3
Duloxetine hydrochloride enteric-coated capsule	0	118	Sodium valproate sustained-release tablet	0	110
	1	1		1	9
Fenofibrate capsule	0	115	Sodium valproate tablet	0	105
	1	4		1	14
Finasteride tablet	0	116	Valsartan capsule	0	117
	1	3		1	2
Fluoxetine hydrochloride capsule	0	116	Zopiclone tablet	0	97
	1	3		1	22

*N* denotes the number of patients. Categories 0 and 1 represent without drug and with drug, respectively.

### 3.2 Modeling

In the final model, weight and concomitant medication of fluoxetine were included as covariants. The aripiprazole dosage form, or the physiological and biochemical indices, or other concomitant medications were not included in the final model.

Therefore, the final model for aripiprazole in patients with schizophrenia was as follows, Equations 6 and 7:


(6) $$CL/F=3.23\times {\left(weight/70\right)}^{0.75}\times \left(1-0.286\times FLU\right)$$

(7) V/F=157×(weight/70)

where CL/F is the apparent oral clearance; *V*/F is the apparent volume of distribution; and FLU denotes fluoxetine. For patients taking fluoxetine, FLU was 1; otherwise, FLU was 0.

### 3.3 Evaluation

**Figure 1:**
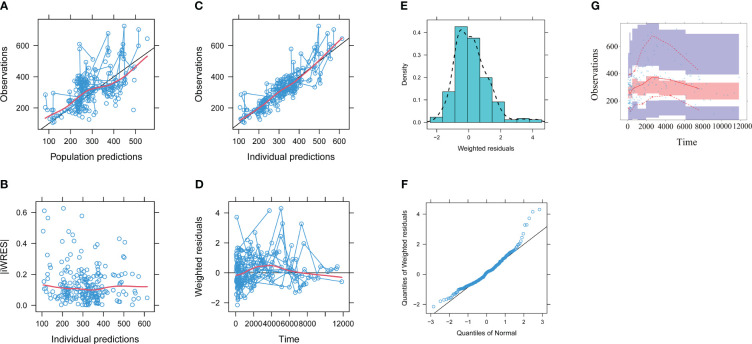
Model evaluation. **(A)** Observations vs. population predictions. **(B)** Absolute value of the weighted residuals of individuals (│iWRES│) vs. individual predictions. **(C)** Observations vs. individual predictions. **(D)** Weighted residuals vs. time. **(E)** Density vs. weighted residuals. **(F)** Quantiles of weighted residuals vs. quantiles of normal. **(G)** Visual predictive check (VPC) of the model. The *middle solid line* represents the median of the prediction-corrected concentrations. The *lower* and *upper dashed lines* are the 2.5th and 97.5th percentiles of the prediction-corrected concentrations.

**Figure 2:**
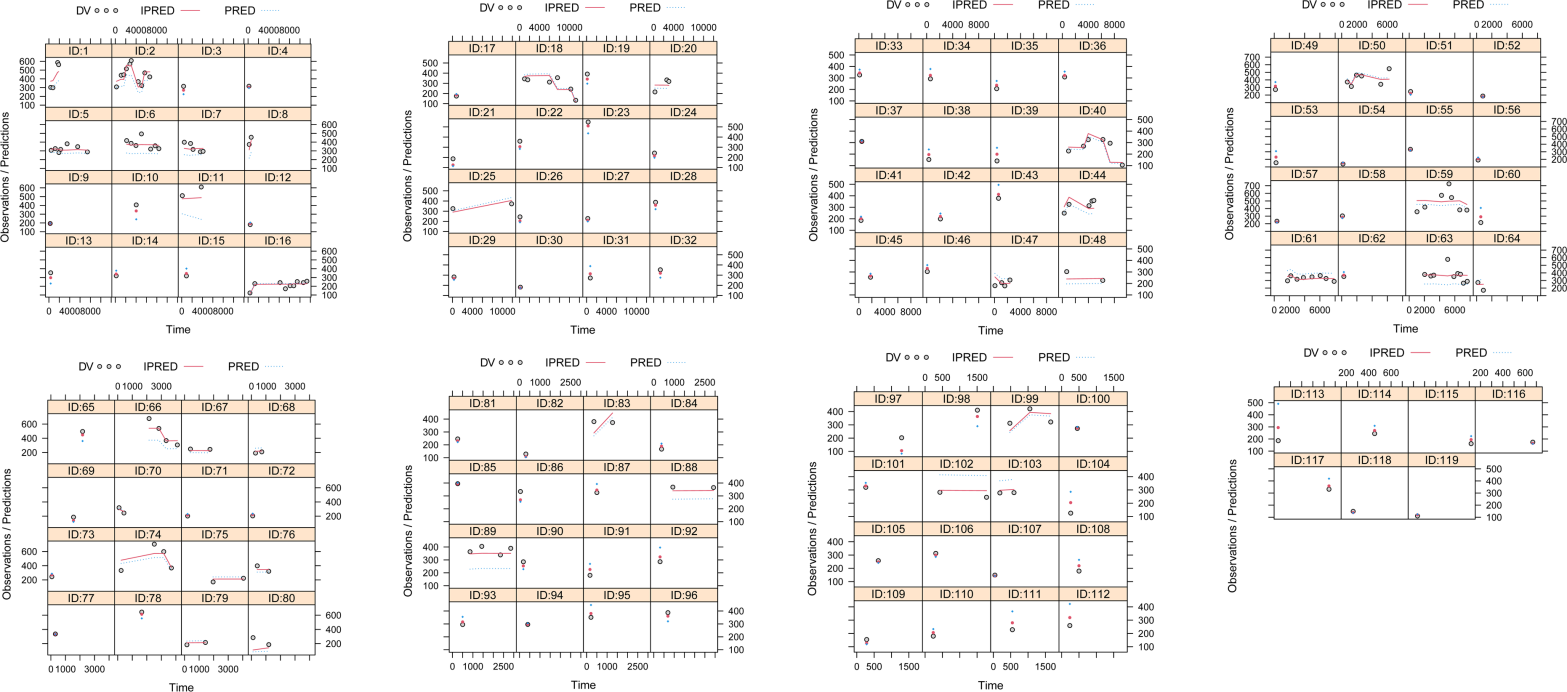
Individual plots. *ID*, patient ID number; *DV*, measured concentration value; *IPRED*, individual predictive value; *PRED*, population predictive value.

The model evaluation is shown in **Figure 1**. **Figures 1A–G** represent the observations vs. population predictions, the │iWRES│ vs. individual predictions, the observations vs. individual predictions, the weighted residuals vs. time, the density vs. weighted residuals, the quantiles of weighted residuals vs. the quantiles of normal, and the VPC of the model, respectively. It was found that the distribution of the final model was normal and that most of the observed aripiprazole concentrations were within the 95% prediction intervals from the simulation data, showing that the aripiprazole concentrations were well predicted by the final model. **Figure 2** displays the individual plots. From a clinical point of view, the predictability value is acceptable. **Table 3** shows the parameter estimates of the final model and bootstrap validation, where the median values of 1,000 bootstraps were close to the respective parameter values of final model, indicating that the final model is reliable and accurate.

**Table 3:** Parameter estimates of the final model and bootstrap validation.

Parameter	Estimate	SE (%)	Bootstrap			Bias (%)
			Median	95% Confidence interval		
CL/F (L/h)	3.23	2.8	3.22	3.04–3.40		−0.31
V/F (L)	157	15.3	160	113–235		1.91
Ka (h^-1^)	1.06 (fixed)	–	–	–		–
***θ***_FLU_	−0.286	6.6	−0.286	−0.383 to −0.239		0
***ω***_CL/F_	0.233	12.2	0.233	0.172–0.291		0
***σ***_1_	0.123	25.6	0.120	0.043–0.176		−2.44
***σ***_2_	49.498	19.4	49.498	14.614–64.440		0

The 95% confidential interval is displayed as the 2.5th–97.5th percentile range of the bootstrap estimates. Bias is the prediction error, calculated as: Bias = (Median − Estimate)/Estimate × 100%.

CL/F, apparent oral clearance (in liters per hour); V/F, apparent volume of distribution (in liters); K_a_, absorption rate constant (per hour); *θ*_FLU_, coefficient of fluoxetine; *ω*_CL/F_, inter-individual variability of CL/F; *σ*_1_, residual variability, proportional error; *σ*_2_, residual variability, additive error.

### 3.4 Simulation

**Figure 3:**
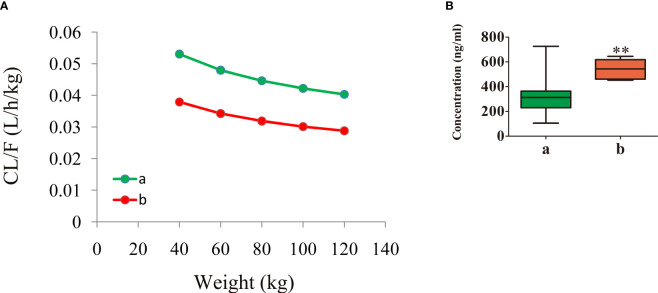
Aripiprazole apparent clearance rate and measured concentrations in patients with schizophrenia. **(A)** Aripiprazole apparent clearance rate. **(B)** Measured aripiprazole concentrations. **(A)** Without fluoxetine. **(B)** With fluoxetine. ^**^p < 0.01 vs. patients with schizophrenia without fluoxetine.

**Figure 3** shows the aripiprazole apparent clearance rate and the measured concentrations in patients with schizophrenia. **Figures 3A, B** display the aripiprazole apparent clearance rate and the measured aripiprazole concentrations, respectively. Line a in the figure denotes patients with schizophrenia without fluoxetine, while line b denotes patients with schizophrenia with fluoxetine. Under the same weight, the aripiprazole clearance rates were 0.714:1 in patients with or without fluoxetine, respectively. Compared with those in schizophrenia patients without fluoxetine, the aripiprazole concentrations in patients with fluoxetine were higher (*p* < 0.01).

**Figure 4:**
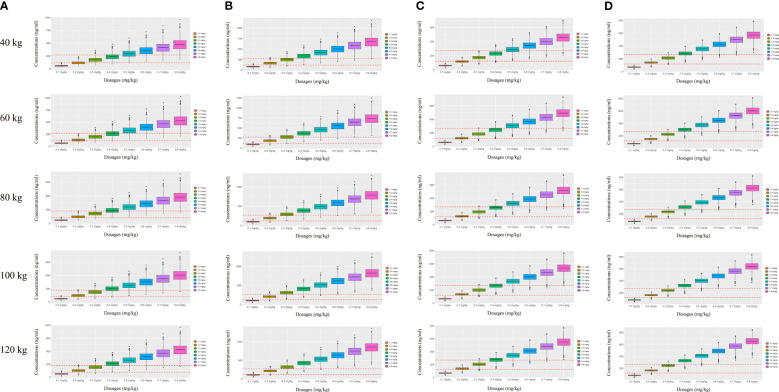
Simulated aripiprazole concentrations. **(A, B)** Simulated aripiprazole concentrations of the once-daily aripiprazole regimen without fluoxetine **(A)** and with fluoxetine **(B)**. **(C, D)** Simulated aripiprazole concentrations of the twice-daily aripiprazole regimen without fluoxetine **(C)** and with fluoxetine **(D)**. The *lower* and *upper red dashed lines* denote 120 and 270 ng/ml, respectively.

**Figure 5:**
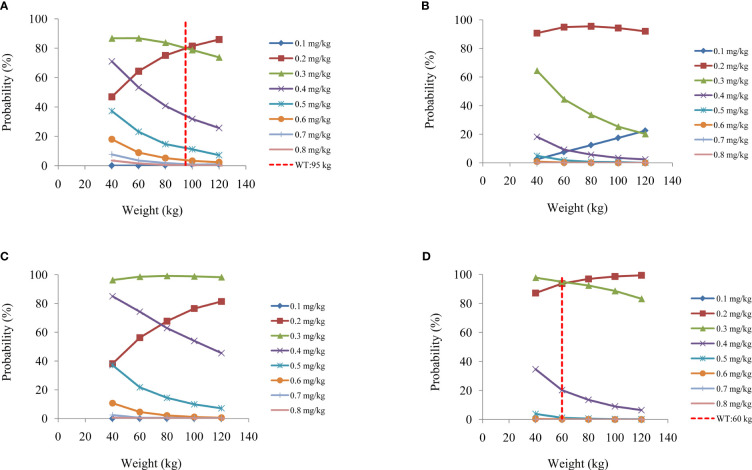
Probability to achieve the target concentrations. **(A, B)** Once-daily aripiprazole regimen without fluoxetine **(A)** and with fluoxetine **(B)**. **(C, D)** Twice-daily aripiprazole regimen without fluoxetine **(C)** and with fluoxetine **(D)**.

On the strength of the final model, the present study simulated four different scenarios: a) a once-daily aripiprazole regimen without fluoxetine; b) a once-daily aripiprazole regimen with fluoxetine; c) a twice-daily aripiprazole regimen without fluoxetine; and d) a twice-daily aripiprazole regimen with fluoxetine. The simulated aripiprazole concentrations of the once-daily aripiprazole regimen without and with fluoxetine and the twice-daily aripiprazole regimen without and with fluoxetine are shown in **Figures 4A–D**, respectively. The lower and upper red dashed lines indicate the treatment window ranges from 120 to 270 ng/ml. **Figure 5** shows the probability to achieve the target concentrations, with **Figures 5A–D** showing the once-daily aripiprazole regimen without and with fluoxetine and the twice-daily aripiprazole regimen without and with fluoxetine, respectively.

Based on these results, we made recommendations for the optimal aripiprazole initial dosage in patients with schizophrenia in each scenario, which are shown in **Table 4**. Without fluoxetine, for the once-daily aripiprazole regimen, dosages of 0.3 and 0.2 mg kg^−1^ day^−1^ were recommended for patients with schizophrenia weighing 40–95 and 95–120 kg, respectively, with the probabilities to achieve the target concentrations for the 0.3- and 0.2-mg kg^−1^ day^−1^ dosages being 80.1%–86.8% and 80.1%–85.9%, respectively. For the twice-daily aripiprazole regimen, a dosage of 0.3 mg kg^−1^ day^−1^ was recommended for patients with schizophrenia weighing 40–120 kg, with the probability to achieve the target concentrations for this dosage being 96.3%–99.2%. With fluoxetine, for the once-daily aripiprazole regimen, a dosage of 0.2 mg kg^−1^ day^−1^ was recommended for patients with schizophrenia weighing 40–120 kg, with the probability to achieve the target concentration for this dosage being 90.7%–95.5%. For the twice-daily aripiprazole regimen, dosages of 0.3 and 0.2 mg kg^−1^ day^−1^ were recommended for patients with schizophrenia weighing 40–60 and 60–120 kg, respectively, with the probabilities to achieve the target concentrations for the 0.3- and 0.2-mg kg^−1^ day^−1^ dosages being 94.3%–97.8% and 94.3%–99.4%, respectively.

**Table 4:** Initial dosage recommendations of aripiprazole in schizophrenic patients without or with fluoxetine.

Without fluoxetine			With fluoxetine		
Body weight (kg)	Dose (mg kg^−1^ day^−1^)	Probability to achieve the target concentrations (%)	Body weight (kg)	Dose (mg kg^−1^ day^−1^)	Probability to achieve the target concentrations (%)
Once a day			Once a day		
40–95	0.3	80.1–86.8	40–120	0.2	90.7–95.5
95–120	0.2	80.1–85.9			
Split evenly into two doses a day			Split evenly into two doses a day		
40–120	0.3	96.3–99.2	40–60	0.3	94.3–97.8
			60–120	0.2	94.3–99.4

## 4 Discussion

In the course of clinical drug combinations, all drugs that might inhibit or activate the enzymes that have profound effects on the metabolism or transport of aripiprazole could have potential drug–drug interactions with aripiprazole, further affecting its concentration and initial dosage optimization in patients with schizophrenia. The main metabolic pathways of aripiprazole in the liver include dehydrogenation, hydroxylation, and *N*-dealkylation via the CYP2D6 and CYP3A4 enzymes (17). However, aripiprazole is metabolized to a lesser extent by the CYP3A4 enzyme, implying that CYP3A4 does not have an appreciable impact on the pharmacokinetics of aripiprazole (17–19).

As is well known, in the treatment of patients with schizophrenia using aripiprazole, TDM is often employed to determine the aripiprazole concentration as it is closely related to the efficacy of treatment and the occurrence of adverse reactions. Previously, on the strength of the 2017 Arbeitsgemeinschaft für Neuropsychopharmakologie und Pharmakopsychiatrie (AGNP)-TDM expert group consensus guidelines for the TDM recommendation for aripiprazole, the treatment window for this drug is 100–350 ng/ml (20). However, Hart et al. presented a prototypical meta-analysis of the relationships between the blood levels of aripiprazole, its target engagement in the human brain, and the clinical effects and side effects in patients with schizophrenia and related disorders, suggesting that the treatment window of aripiprazole for schizophrenia is 120–270 ng/ml (16). The study of Hart et al. further refined the reference range of aripiprazole in patients with schizophrenia and provided a reference for realizing the individualized administration of aripiprazole in this group of patients (16).

Clinically, the subsequent dosage of aripiprazole can be adjusted based on the TDM feedback to achieve individualized administration needs. However, there are no TDM values available for initial dosage administration. Fortunately, population pharmacokinetics combined with Monte Carlo simulations can identify potential drug–drug interactions and optimize the initial dosage schedules (21–23). Therefore, in this study, we combined population pharmacokinetics and Monte Carlo simulations to investigate the drug–drug interactions and the initial dosage optimization of aripiprazole in patients with schizophrenia.

A total of 119 patients with schizophrenia treated with aripiprazole were included to build an aripiprazole population pharmacokinetic model using NONMEM. The physiological and biochemical markers, as well as details of the drug combinations, in patients with schizophrenia were collected and analyzed as potential covariates. In the final model, the weight and the concomitant medication of fluoxetine influenced aripiprazole clearance. This is mainly due to the metabolism of aripiprazole via CYP2D6. Fluoxetine can inhibit CYP2D6, thereby reducing aripiprazole metabolism, which affects its blood concentration and dosage. Under the same weight, the aripiprazole clearance rates were 0.714:1 in patients with or without fluoxetine, respectively. Compared with schizophrenia patients without fluoxetine, the aripiprazole concentrations in those with fluoxetine were higher.

In addition, the present study simulated four different scenarios: a once-daily aripiprazole regimen without fluoxetine, a once-daily aripiprazole regimen with fluoxetine, a twice-daily aripiprazole regimen without fluoxetine, and a twice-daily aripiprazole regimen with fluoxetine. Without fluoxetine, for the once-daily aripiprazole regimen, dosages of 0.3 and 0.2 mg kg^−1^ day^−1^ were recommended for patients with schizophrenia weighing 40–95 and 95–120 kg, respectively, while for the twice-daily aripiprazole regimen, 0.3 mg kg^−1^ day^−1^ was recommended for those weighing 40–120 kg. With fluoxetine, for the once-daily aripiprazole regimen, 0.2 mg kg^−1^ day^−1^ was recommended for patients with schizophrenia weighing 40–120 kg, while for the twice-daily aripiprazole regimen, 0.3 and 0.2 mg kg^−1^ day^−1^ were recommended for those weighing 40–60 and 60–120 kg, respectively.

However, as our data were derived from real-world clinical sparse trough concentrations, the predictive ability was objectively deficient. In the future, we will design prospective aripiprazole intensive sampling sites to further carry out relevant studies.

## 5 Conclusion

This is the first time the effects of fluoxetine on aripiprazole via drug–drug interactions were investigated. We recommend the optimal aripiprazole initial dosage in patients with schizophrenia based on population pharmacokinetics. In schizophrenia patients with concomitant medication of fluoxetine, the aripiprazole dosage might need to be adjusted.

## Data availability statement

The original contributions presented in the study are included in the article/supplementary material. Further inquiries can be directed to the corresponding authors.

## Ethics statement

The studies involving humans were approved by Research Ethics Committee of the Xuzhou Oriental Hospital Affiliated to Xuzhou Medical University. The studies were conducted in accordance with the local legislation and institutional requirements. The ethics committee/institutional review board waived the requirement of written informed consent for participation from the participants or the participants’ legal guardians/next of kin because the data were collected retrospectively without patient identifiers.

## Author contributions

CZ: Supervision, Software, Methodology, Data curation, Writing – review & editing. LJ: Supervision, Methodology, Writing – review & editing. KH: Software, Methodology, Writing – review & editing. Y-JZ: Software, Methodology, Writing – review & editing. JH: Software, Methodology, Writing – review & editing. JC: Software, Methodology, Writing – review & editing. B: Software, Methodology, Writing – review & editing. BD: Software, Methodology, Writing – review & editing. H-ZS: Software, Methodology, Data curation, Writing – review & editing. S-MH: Visualization, Validation, Software, Resources, Project administration, Investigation, Formal analysis, Conceptualization, Writing – review & editing. T-TY: Visualization, Resources, Methodology, Data curation, Writing – review & editing. XC: Validation, Supervision, Software, Resources, Methodology, Investigation, Funding acquisition, Formal analysis, Data curation, Conceptualization, Writing – review & editing. D-DW: Visualization, Validation, Supervision, Software, Resources, Project administration, Methodology, Investigation, Funding acquisition, Formal analysis, Data curation, Conceptualization, Writing – original draft.
